# Supplementary material for: Phacoemulsification combined with goniosynechialysis versus phacoemulsification alone for patients with primary angle‑closure disease: A meta-analysis
Source: PLoS One. 2024 Feb 2;19(2):e0296849. doi: 10.1371/journal.pone.0296849 (PMC10836673; doi:10.1371/journal.pone.0296849)
Supplement: Attachment — Submitted filename: PONE-D-23-24472_reviewerV1.pdf [file pone.0296849.s002.pdf]

PLOS ONE

Phacoemulsification combined with goniosynechialysis versus phacoemulsification alone for patients with primary angle-closure disease: a meta-analysis

--Manuscript Draft--

**Manuscript Number:**	PONE-D-23-24472
**Article Type:**	Research Article
**Full Title:**	Phacoemulsification combined with goniosynechialysis versus phacoemulsification alone for patients with primary angle-closure disease: a meta-analysis
**Short Title:**	PE combined with GSL versus PE alone for PACD
**Corresponding Author:**	Haiqing Bai<br>The Affiliated Hospital of Qingdao University, Qingdao, China<br>Qingdao, CHINA
**Keywords:**	Phacoemulsification (PE); Goniosynechialysis (GSL); Viscogonioplasty (VGP); Primary Angle-closure Disease (PACD); meta-analysis
**Abstract:**	This meta-analysis aims to systematically compare the efficacy between phacoemulsification (PE) combined with goniosynechialysis (GSL) and PE alone for primary angle-closure disease (PACD) patients. All the data were searched from the PubMed, EMBASE and the Cochrane Library. The Cochrane Handbook was used to evaluate the quality of the included studies. Additionally, this meta-analysis was performed by using the Revman 5.4 software. Nine randomized controlled trials (RCTs) were included in this study. Compared with PE alone group, PE+GSL could result significant reduction in the IOP (MD, 1.81; p=0.002). In the instrumental subgroup, also more reduction of IOP was shown in the PE+GSL group (MD, 2.11; p=0.02). In the viscogonioplasty (VGP) subgroup, there was not no statistical difference between PE alone group and PE+GSL group (MD, 1.53; p=0.11). Also, more reduction of peripheral anterior synechiae (PAS) was shown in the PE+GSL group (MD,59.15; p<0.00001). For the change in angle open distance (AOD)500, AOD 750, trabecular-iris space (TISA)500, number of glaucoma medications and best corrected visual acuity (BCVA), there was no difference between two groups (p=0.25, 0.35, 0.17, 0.56, 0.08). For TSIA 750, more improvement was shown in the PE+GSL group (p<0.00001). Instrumental separation had better effect on lowering IOP when it combined with PE. Both instrumental separation and VGP could reduce postoperative PAS. The operation of GSL has no obvious effect on postoperative vision.
**Order of Authors:**	Lin Yao
	Haitao Wang
	Yunxiao Wang
	Pengpeng Zhao
	Haiqing Bai
**Additional Information:**	
**Question**	**Response**
**Financial Disclosure**<br><br>Enter a financial disclosure statement that describes the sources of funding for the work included in this submission. Review the submission guidelines for detailed requirements. View published research articles from *PLOS ONE* for specific examples.	NO

Powered by Editorial Manager® and ProduXion Manager® from Aries Systems Corporation

This statement is required for submission and **will appear in the published article** if the submission is accepted. Please make sure it is accurate.<br><br>**Unfunded studies**<br>Enter: *The author(s) received no specific funding for this work.*<br><br>**Funded studies**<br>Enter a statement with the following details:<br>• Initials of the authors who received each award<br>• Grant numbers awarded to each author<br>• The full name of each funder<br>• URL of each funder website<br>• Did the sponsors or funders play any role in the study design, data collection and analysis, decision to publish, or preparation of the manuscript?<br>• **NO** - Include this sentence at the end of your statement: *The funders had no role in study design, data collection and analysis, decision to publish, or preparation of the manuscript.*<br>• **YES** - Specify the role(s) played.<br><br>* typeset	
**Competing Interests**<br><br>Use the instructions below to enter a competing interest statement for this submission. On behalf of all authors, disclose any competing interests that could be perceived to bias this work—acknowledging all financial support and any other relevant financial or non-financial competing interests.<br><br>This statement is required for submission and **will appear in the published article** if the submission is accepted. Please make sure it is accurate and that any funding sources listed in your Funding Information later in the submission form are also declared in your Financial Disclosure statement.<br><br>View published research articles from *PLOS ONE* for specific examples.	The authors have declared that no competing interests exist.

Powered by Editorial Manager® and ProduXion Manager® from Aries Systems Corporation

**NO authors have competing interests**<br><br>Enter: *The authors have declared that no competing interests exist.*<br><br>**Authors with competing interests**<br><br>Enter competing interest details beginning with this statement:<br><br>*I have read the journal's policy and the authors of this manuscript have the following competing interests: [insert competing interests here]*<br><br>* typeset	
**Ethics Statement**<br><br>Enter an ethics statement for this submission. This statement is required if the study involved:<br><br>• Human participants<br>• Human specimens or tissue<br>• Vertebrate animals or cephalopods<br>• Vertebrate embryos or tissues<br>• Field research<br><br>Write "N/A" if the submission does not require an ethics statement.<br><br>General guidance is provided below. Consult the submission guidelines for detailed instructions. **Make sure that all information entered here is included in the Methods section of the manuscript.**	N/A

**Format for specific study types**<br><br>**Human Subject Research (involving human participants and/or tissue)**<br>• Give the name of the institutional review board or ethics committee that approved the study<br>• Include the approval number and/or a statement indicating approval of this research<br>• Indicate the form of consent obtained (written/oral) or the reason that consent was not obtained (e.g. the data were analyzed anonymously)<br><br>**Animal Research (involving vertebrate animals, embryos or tissues)**<br>• Provide the name of the Institutional Animal Care and Use Committee (IACUC) or other relevant ethics board that reviewed the study protocol, and indicate whether they approved this research or granted a formal waiver of ethical approval<br>• Include an approval number if one was obtained<br>• If the study involved *non-human primates*, add *additional details* about animal welfare and steps taken to ameliorate suffering<br>• If anesthesia, euthanasia, or any kind of animal sacrifice is part of the study, include briefly which substances and/or methods were applied<br><br>**Field Research**<br><br>Include the following details if this study involves the collection of plant, animal, or other materials from a natural setting:<br>• Field permit number<br>• Name of the institution or relevant body that granted permission	
**Data Availability**<br><br>Authors are required to make all data underlying the findings described fully available, without restriction, and from the time of publication. PLOS allows rare exceptions to address legal and ethical concerns. See the PLOS Data Policy and FAQ for detailed information.	Yes - all data are fully available without restriction

A Data Availability Statement describing where the data can be found is required at submission. Your answers to this question constitute the Data Availability Statement and **will be published in the article**, if accepted.<br><br>**Important:** Stating 'data available on request from the author' is not sufficient. If your data are only available upon request, select 'No' for the first question and explain your exceptional situation in the text box.<br><br>Do the authors confirm that all data underlying the findings described in their manuscript are fully available without restriction?	
**Describe where the data may be found in full sentences. If you are copying our sample text, replace any instances of XXX with the appropriate details.**<br><br>• If the data are **held or will be held in a public repository**, include URLs, accession numbers or DOIs. If this information will only be available after acceptance, indicate this by ticking the box below. For example: *All XXX files are available from the XXX database (accession number(s) XXX, XXX.)*.<br>• If the data are all contained **within the manuscript and/or Supporting Information files**, enter the following: *All relevant data are within the manuscript and its Supporting Information files.*<br>• If neither of these applies but you are able to provide **details of access elsewhere**, with or without limitations, please do so. For example:<br><br>*Data cannot be shared publicly because of [XXX]. Data are available from the XXX Institutional Data Access / Ethics Committee (contact via XXX) for researchers who meet the criteria for access to confidential data.*<br><br>*The data underlying the results presented in the study are available from (include the name of the third party*	All relevant data are within the manuscript and its Supporting Information files.

Specify tha

and contact information or URL).<br>• This text is appropriate if the data are owned by a third party and authors do not have permission to share the data.<br><br>* typeset	
Additional data availability information:	

Powered by Editorial Manager® and ProduXion Manager® from Aries Systems Corporation

Manuscript
Click here to access/download;Manuscript;manuscript - revised0819.docx

Phacoemulsification combined with goniosynechialysis versus phacoemulsification alone for patients with primary angle‑closure disease: a meta-analysis

Running Head：PE combined with GSL versus PE alone for PACD

Authors:

Lin Yao[a]*, Haitao Wang[b]*, Yunxiao Wang[b]*, Pengpeng Zhao[a], Haiqing Bai[b]

a, Qingdao Aier Eye Hospital, Qingdao, China

b, Department of Ophthalmology, The Affiliated Hospital of Qingdao University, Qingdao, China

* These authors contributed equally to this work as first authors.

Corresponding Author: Haiqing Bai

Department of Ophthalmology, The Affiliated Hospital of Qingdao University, Qingdao, China

Address：16th, Jiangsu Road, Qingdao, 266003, China

Mobile phone: 86-18661806365

Email: haiqing_bai@126.com＆haiqingbai@qdu.edu.cn

Competing Interests:

The authors have declared that no competing interests exist.

No financial disclosures related to the contents of the manuscript.

There was non-financial support for the review.

Abstract

This meta-analysis aims to systematically compare the efficacy between phacoemulsification (PE) combined with goniosynechialysis (GSL) and PE alone for primary angle-closure disease (PACD) patients. All the data were searched from the PubMed, EMBASE and the Cochrane Library. The Cochrane Handbook was used to

There are many different scales. Which one was used?

evaluate the quality of the included studies. Additionally, this meta-analysis was performed by using the Revman 5.4 software. Nine randomized controlled trials (RCTs) were included in this study. Compared with PE alone group, PE+GSL could result significant reduction in the IOP (MD, 1.81; p=0.002). In the instrumental

Important to elucidadte the result of the metanalysys, with RR)

subgroup, also more reduction of IOP was shown in the PE+GSL group (MD, 2.11; p=0.02). In the viscogonioplasty (VGP) subgroup, there was not no statistical difference between PE alone group and PE+GSL group (MD, 1.53; p=0.11). Also, more reduction of peripheral anterior synechiae (PAS) was shown in the PE+GSL group (MD,59.15; p<0.00001). For the change in angle open distance (AOD)500, AOD 750, trabecular-iris space (TISA)500, number of glaucoma medications and best corrected visual acuity (BCVA), there was no difference between two groups (p=0.25, 0.35, 0.17, 0.56, 0.08). For TSIA 750, more improvement was shown in the PE+GSL group (p<0.00001). Instrumental separation had better effect on lowering IOP when it combined with PE. Both instrumental separation and VGP could reduce postoperative PAS. The operation of GSL has no obvious effect on postoperative vision. TISA?)

Keywords: Phacoemulsification (PE); Goniosynechialysis (GSL); Viscogonioplasty (VGP); Primary Angle-closure Disease (PACD); Meta-analysis Introduction

Primary angle-closure glaucoma (PACG) usually can cause irreversible damage to vision. PACG is worldly estimated to affect 15 million people. It is more common amongst Asians, especially Chinese [1-3]. The blockage of the aqueous drainage due to the peripheral anterior synechiae (PAS), which finally results in the raising

intraocular pressure (IOP) [4]. Crowed anterior chamber and iridotrabecular meshwork contact are the predisposition for PAS [4].

In eyes with angle closure, lens extraction by phacoemulsificatoin (PE) can deepen the anterior chamber and reverse the mechanism of the angle-crowding, meanwhile, decrease the iridotrabecular contact, widen the angle and improve aqueous outflow facility. Therefore, PE can lower the IOP for patients with PACG [5,6]. Furthermore, some studies have shown that the lowering IOP depended on the reduction of PAS [7,8]. Goniosynechialysis (GSL) which can be performed combined with PE, is a surgical technique to break the PAS [9-14]. It involves the physical separation of PAS by using surgical instrument under direct visualization with goniolens or by cohesive viscoelastic without direct visualization. The latter technique is also called viscogonioplasty (VGP) [6,15,16].

Recent years, many studies have demonstrated that both PE combined with GSL (PE+GSL) and PE alone can effectively lower the IOP for patients with primary angle-closure (PAC) or PACG. Correspondingly, some of these studies compared these two surgeries [6,9-16]. The primary objective of this meta-analysis is to compare the efficacy between PE+GSL and PE alone for patients with PAC or PACG.

Materials and Methods

This meta-analysis of prospective randomized clinical trials (RCTs) was performed according to the Preferred Items for Systematic Reviews and Meta-Analyses (PRISMA) guidelines.

There are specific guidelines to describe methodology. Authors should state that only RCT were included

Methods of Literature search

LILACS is also an important database. Why it was not used?)

Computer databases including PubMed, EMBASE and Cochrane Library were searched from the year 2000 to January 2023. Year 2000 was selected to be consistent with the way PE surgeries were performed. Medical Subject Headings (MeSH) and free words closely related with PE and GSL were used. (((phaco*[Title/Abstract]) OR

Please make this clearer.

(phacoemulsification[Title/Abstract])) OR (phako*[Title/Abstract])) AND ((((viscogonioplasty[Title/Abstract]) OR (goniosynechialysis[Title/Abstract])) OR (GSL[Title/Abstract])) OR (VGP[Title/Abstract])) was used for searching from the PubMed.

Inclusion criteria

(1) RCTs.

(2) Patient populations with PAC or PACG.

(3) All eyes were treated with PE with or without GSL.

(4) At least one primary or secondary outcome reported.

(5) Follow-up time was more than one month.

(6) Studies published in English.

Exclusion criteria

(1) Non-randomized studies.

(2) Cohort studies, case report, reviews and editorial.

(3) Studies without outcomes of interest.

Data extraction

Two reviewers (LY and HB) independently extracted data using the pre-established extraction tables, including the following: (1) Basic characteristics of the study, including the name of the first author, year of publication, and follow-up time (2) Basic characteristics of the patients, including the mean age and standard deviation

I suppose they mean demographic data. Important ethnic characteristics

(SD) (3) Baseline and post-operative characteristics such as IOP, range of PAS, AOD, TISA, number of glaucoma medications and BCVA. If consensus was not achieved by these two reviewers, the third reviewer would be intervened to provide a decision

[17-20]. Specify that papers with different languages would be excluded

Outcome measures Digite o texto aqui

The primary outcomes were mean change in IOP and PAS. The secondary outcomes were angle open distance (AOD) at 500 and 750μm, trabecular-iris space (TISA) at 500 and 750μm, mean change in number of glaucoma medications from baseline and best corrected visual acuity (BCVA).

Qualitative assessment Cochrane Quality scale described in the Handbook

The quality of the included studies was assessed by the Cochrane Handbook, including 6 items: random sequence generation, allocation concealment, blinding of participants and personnel, blinding of outcome assessment, incomplete outcome data, selective reporting, and other biases. Two reviewers determined the risk of bias which had three options (low, high, and unclear) [21]. If consensus was not achieved by these two reviewers, the third reviewer would be intervened to provide a decision. When necessary, we contacted the authors of the studies to obtain the full text or related information for an accurate assessment [17-20].

Statistical analysis

Review Manager (version 5.4; Cochrane Collaboration) was used for data analysis. The statistical heterogeneity of the included studies was test by the Cochrane I^2 test. A random-effect model was applied. Mean difference (MD) with a 95% confidence interval (CI) was used to estimate the effectiveness for continuous variable data [17-20]. And Relative Risk for PAS formation wasn't calculated?

Subgroup analyses were performed according to whether the GSL performed by using surgical instrument (instrumental subgroup) or viscoelastic (VGP subgroup).

Results

A total of 96 studies were retrieved. Finally, 9 RCTs were included in this

please specify in Methods (subgroup analysis, which are this instruments), how is the procedure done)

meta-analysis. The basic characteristics of the included studies were shown in Table 1. There were 237 patients in the PE+GSL group and 229 patients in the PE alone group. In six studies, surgical instrument was used to physically break the PAS under gonioscopic visualization in the PE+GSL group. In other three studies, patients in the PE+GSL group were received VGP, a cohesive viscoelastic was injected near the angle following intraocular lens (IOL) implantation to break the PAS. The literature screening process was shown in Figure 1.

Methodological quality evaluation

The results of the methodological evaluation according to the Cochrane Handbook were shown in Figure 2.

Please detail the quality of studies that was presented in Figure 2. The majority of studies were considered of high quality, because they fullfil the criteria descreibed in the Cochrane Scale, correct?

Efficacy analysis

A statistically significant mean difference in IOP was seen when studies were compared, in favor to PE+GSL group. This difference was only detected when instruments under direct view was used (descr
When viscodissection was used, this difference was not stattistically significant.

Mean change in IOP from baseline

All nine studies reported changes in IOP from baseline. More reduction of IOP was shown in the PE+GSL group (MD, 1.81; p=0.002). In the instrumental subgroup, also more reduction of IOP was shown in the PE+GSL group (MD, 2.11; p=0.02). However, in the VGP subgroup, there was not statistical difference between the PE+GSL group and PE alone group (MD, 1.53; p=0.11) (Figure 3).

Some studies have strange numbers of Mena final IOP, please check

Please describe heterogenity observed

Figures have to have description, specifing the comparison

Mean change in PAS

Heterogeneity should be described and discussed for all the comparisons

Six studies reported changes in PAS. The overall MD was 59.15 (95%CI, 36.28 to 82.03), compared with PE alone group more reduction of PAS was shown in the PE+GSL group (p<0.00001). As well, in the instrumental subgroup and VGP subgroup, more reduction of PAS was shown in the PE+GSL group (MD, 53.32, 66.00; 95%CI, 5.96 to 100.67, 43.13 to 88.88; p=0.03, <0.00001) (Figure 4).

Mean change in number of glaucoma medications

Eight studies reported the change in number of glaucoma medications before and after

surgery. The overall MD was 0.08 (95%CI, -0.19 to 0.35), and there was no statistical difference between the PE+GSL group and PE alone group (p=0.56). As well, in the instrumental subgroup and VGP subgroup analysis, there was not statistical difference between the PE+GSL group and PE alone group (MD, 0.05, 0.20; 95%CI, -0.32 to 0.42, -0.12 to 0.52; p=0.79, 0.22) (Figure 5).

Mean change in AOD 500 and AOD 750

Three studies reported the change in AOD before and after surgery. The overall MD was 0.16 and 0.21 (95%CI, -0.12 to 0.45, -0.23 to 0.66), and there was no statistical difference between the PE+GSL group and PE alone group in both AOD 500 and 750 (p=0.25 and 0.35). As well, in the instrumental subgroup and VGP subgroup, there was not statistical difference between the PE+GSL group and PE alone group (AOD 500, MD, 0.24, 0.03; 95%CI, -0.08 to 0.55, -0.02 to 0.09; AOD 750, MD, 0.30, 0.03; 95%CI, -0.29 to 0.89, -0.04 to 0.10; p=0.31,0.44) (Figure 6,7).

Mean change in TISA 500 and TISA 750

Four studies reported the change in TISA 500 and five studies reported the change in TISA 750. For TISA 500, there was no statistical difference between the PE+GSL group and PE alone group (MD, p=0.17). As well, in the instrumental subgroup and VGP subgroup, there was no statistical difference between the PE+GSL group and PE alone group (MD, 0.07, 0.02; 95%CI, -0.03 to 0.18, -0.01 to 0.04; p=0.16,0.13) (Figure 8). For TSIA750, more improvement was shown in the PE+GSL group (MD, 0.10; p<0.00001). As well, in the instrumental subgroup and VGP subgroup, more improvement was shown in the PE+GSL group (MD, 0.17, 0.03; 95%CI, 0.15 to 0.19, 0.01 to 0.05; p<0.00001, p=0.008) (Figure 9).

Mean change in BCVA from baseline

Six studies reported changes in BCVA from baseline. The overall MD was 0.03 (95%CI, -0.03 to 0.09), and there was no statistical difference between the PE+GSL

group and PE alone group (p=0.29). As well, in the instrumental subgroup and VGP subgroup, there was not statistical difference between two groups (MD, 0.05, 0; 95%CI, -0.03 to 0.13, -0.09 to 0.09; p=0.21,0.96) (Figure 10).

Discussion

According to the International Society of Geographic and Epidemiologic Ophthalmology (ISGEO) classification, PAC had led to significant glaucomatous damage to the optic nerve would be defined as PACG [22]. Therefore, both PAC and PACG should be classified as primary angle-closure disease (PACD). In our meta-analysis, seven from nine included RCTs focused on PACG [6,9,11,13-16], and other two RCTs focused on PAC [10,12].

Previous studies proved that both PE+GSL and PE alone could effectively reduce the IOP of patients with PACD [6,9-16]. Therefore, the potential different effect on lowering IOP derived from PE+GSL and PE alone was the primary issue of concern. Most of the included studies concluded that there was no statistical difference in lowering IOP between the PE+GSL and PE alone group [6,9-11,13-16]. However, as all nine included studies were merged into an overall data, the meta-analysis provided that PE+GSL had better effect of lowering IOP compared with PE alone. As well as in the instrumental subgroup, PE+GSL had better effect of lowing IOP. However, in the VGP subgroup, there was no significant difference in reduction of IOP between PE+GSL and PE alone. This was consistent with all the three included studies focused on VGP in our meta-analysis [6,15,16]. Therefore, the combination of VGP in PE may not bring extra effect of lowering IOP.

Important to discuss heterogeneity

Some studies showed that the lowering IOP depended on the change of PAS extent [7,8]. The trabecular meshwork could be exposed again by separating PAS. Furthermore, the separating PAS and possible restoration of trabecular function before irreversible structural changes, which seem to be a logical approach to reduce IOP of PACG patients [23,24]. Previous studies showed that the decreasing of PAS was

significant after performing both PE+GSL and PE alone [10-12, 14-16]. In this meta-analysis, we further analysed the effect of additional GSL in PE on the PAS extent. The change of PAS extent in the PE+GSL group was significantly greater than PE alone group. In the subgroup analysis, both the instrumental subgroup and VGP subgroup showed the similar results. Previous research revealed that mechanical deepening of the anterior chamber with viscoelastic or saline infusion during PE alone may also open some PAS [25]. Obviously, PE+GSL has better effect of less PAS. After all, GSL is specifically used to break PAS. We found both instrumental separation and VGP could decrease PAS. For the effect of lowering IOP, VGP combined with PE did not seem to bring additional effect compared with PE alone. The reasons may be as follows. The trabecular meshwork which behind the PAS may not be functional. Therefore, the mechanical separating PAS may not result in reduction of IOP. Moreover, gonioscopic appearance might hardly reflect the function of the trabecular meshwork. The loss of trabecular cells as well as the irregular

In the authors'opinion. Not sure ifthis is correct to state.

architecture of the trabeculum which might exist in areas away from visible PAS or under the PAS [26]. However, instrumental separation could bring additional lowering IOP. This may be because the mechanical separation by using surgical instrument under direct visualization is clearer and stronger than VGP. In addition to separation of the PAS, the trabecular meshwork may also be pulled during the separation process, which may make the outflow of aqueous easier. ????

There was no significant difference in most parameters of anterior chamber angle (ACA) between the two groups before and after surgery. Only TISA 750 was different between the two groups. Therefore, GSL may not have much influence on ACA.

Eight involved studies revealed that there was no significant difference in reducing glaucoma medications between the PE+GSL group and PE group [9-16]. Our meta-analysis also confirmed that there was no significant difference in reducing glaucoma medications between two groups. Furthermore, subgroup analyses obtained the same result.

Six included studies concluded that there was no significant difference in improvement of BCVA between the PE+GSL group and the PE group [9,11-13,15,16]. Our meta-analysis also confirmed that no significant difference could be found in improvement of BCVA between two groups. In further subgroup analyses, there was also no significant difference in improvement of BCVA. These results illustrated that the operation of GSL may have no effect on the prognosis of postoperative vision.

Conclusions

Both instrumental separation and VGP could reduce postoperative PAS. However, it seems that only instrumental breaking the PAS could bring extra effect of lowering IOP when it combined with PE. And PE combined with or without GSL has no influence on postoperative vision.

Please, revise the mean IOP in the studies, and recalculate Mean Difference in Figure 3

References

1. Foster PJ, Johnson GJ. Glaucoma in China: how big is the problem?. Br J Ophthalmol. 2001;85(11):1277-1282
2. Quigley HA, Broman AT. The number of people with glaucoma worldwide in 2010 and 2020. Br J Ophthalmol. 2006;90(3):262-267.
3. Tham YC, Li X, Wong TY, Quigley HA, Aung T, Cheng CY. Global prevalence of glaucoma and projections of glaucoma burden through 2040: a systematic review and meta-analysis. Ophthalmology. 2014;121(11):2081-2090.
4. Young CEC, Seibold LK, Kahook MY. Cataract surgery and intraocular pressure in glaucoma. Curr Opin Ophthalmol. 2020;31(1):15-22.
5. Hayashi K, Hayashi H, Nakao F, Hayashi F. Effect of cataract surgery on intraocular pressure control in glaucoma patients. J Cataract Refract Surg. 2001;27(11):1779-1786.
6. Varma D, Adams W, Bunce C, Phelan P, Fraser S. Viscogonioplasty in narrow angle glaucoma: a randomized controlled trial. Clin Ophthalmol. 2010;4:1475-1479.
7. Lee YH, Yun YM, Kim SH, Lee EK, Lee JE, Kim CS. Factors that influence intraocular pressure after cataract surgery in primary glaucoma. Can J Ophthalmol. 2009;44(6):705-710.
8. Shams PN, Foster PJ. Clinical outcomes after lens extraction for visually significant cataract in eyes with primary angle closure. J Glaucoma.

2012;21(8):545-550.
9. Angmo D, Shakrawal J, Gupta B, Yadav S, Pandey RM, Dada T. Comparative Evaluation of Phacoemulsification Alone versus Phacoemulsification with Goniosynechialysis in Primary Angle-Closure Glaucoma: A Randomized Controlled Trial. Ophthalmol Glaucoma. 2019;2(5):346-356.
10. Husain R, Do T, Lai J, et al. Efficacy of Phacoemulsification Alone vs Phacoemulsification With Goniosynechialysis in Patients With Primary Angle-Closure Disease: A Randomized Clinical Trial. JAMA Ophthalmol. 2019;137(10):1107-1113.
11. Lee CK, Rho SS, Sung GJ, et al. Effect of Goniosynechialysis During Phacoemulsification on IOP in Patients With Medically Well-controlled Chronic Angle-Closure Glaucoma. J Glaucoma. 2015;24(6):405-409.
12. Rodrigues IA, Alaghband P, Beltran Agullo L, et al. Aqueous outflow facility after phacoemulsification with or without goniosynechialysis in primary angle closure: a randomised controlled study. Br J Ophthalmol. 2017;101(7):879-885.
13. Shao T, Hong J, Xu J, Le Q, Wang J, Qian S. Anterior Chamber Angle Assessment by Anterior-segment Optical Coherence Tomography After Phacoemulsification With or Without Goniosynechialysis in Patients With Primary Angle Closure Glaucoma. J Glaucoma. 2015;24(9):647-655.
14. Tun TA, Baskaran M, Perera SA, Htoon HM, Aung T, Husain R. Swept-source optical coherence tomography assessment of iris-trabecular contact after phacoemulsification with or without goniosynechialysis in eyes with primary angle closure glaucoma. Br J Ophthalmol. 2015;99(7):927-931.
15. Eslami Y, Latifi G, Moghimi S, et al. Effect of adjunctive viscogonioplasty on drainage angle status in cataract surgery: a randomized clinical trial. Clin Exp Ophthalmol. 2013;41(4):368-378.
16. Moghimi S, Latifi G, ZandVakil N, et al. Phacoemulsification Versus Combined Phacoemulsification and Viscogonioplasty in Primary Angle-Closure Glaucoma: A Randomized Clinical Trial. J Glaucoma. 2015;24(8):575-582.
17. Yao L, Zhang M, Zhao Q, Wang S, Wang D, Bai H. Microstent Implantation with Phacoemulsification Versus Phacoemulsification Alone for Patients with Open Angle Glaucoma: A Systematic Review and Meta-Analysis of Randomised Clinical Trials. Semin Ophthalmol. 2022;37(6):749-755.
18. Yao L, Zhang M, Wang D, Zhao Q, Wang S, Bai H. Small Incision Lenticule Extraction (SMILE) and Laser in Situ Keratomileusis (LASIK) Used to Treat Myopia and Myopic Astigmatism: A Systematic Review and Meta-analysis of Randomized Clinical Trials [published online ahead of print, 2022 Jul 31]. Semin Ophthalmol. 2022;1-11.
19. Yao L, Yang X, Jia S, Wang W, Yang X, Bai H. Combined Surgery Versus Phacoemulsification Alone for Patients with Primary Angle-Closure Glaucoma: A meta-analysis [published online ahead of print, 2022 Nov 16]. Semin Ophthalmol. 2022;1-8.
20. Zhao C, Cai C, Ding Q, Dai H. Efficacy and safety of atropine to control myopia progression: a systematic review and meta-analysis. *BMC Ophthalmol*.

2020;20(1):478. Published 2020 Dec 7.
21. Higgins JPT, Thomas J, Chandler J, Cumpston M, Li T, Page MJ, Welch VA (editors). Cochrane Handbook for Systematic Reviews of Interventions version 6.3 (updated February 2022). Cochrane, 2022. Available from www.training.cochrane.org/handbook.
22. Foster PJ, Buhrmann R, Quigley HA, Johnson GJ. The definition and classification of glaucoma in prevalence surveys. Br J Ophthalmol. 2002;86(2):238-242.
23. Teekhasaenee C, Ritch R. Combined phacoemulsification and goniosynechialysis for uncontrolled chronic angle-closure glaucoma after acute angle-closure glaucoma. Ophthalmology. 1999;106(4):669-675
24. Varma D, Adams WE, Phelan PS, Fraser SG. Viscogonioplasty in patients with chronic narrow angle glaucoma. Br J Ophthalmol. 2006;90(5):648-649.
25. Lai JS, Tham CC, Chan JC. The clinical outcomes of cataract extraction by phacoemulsification in eyes with primary angle-closure glaucoma (PACG) and co-existing cataract: a prospective case series. J Glaucoma. 2006;15(1):47-52.
26. Liu CJ, Cheng CY, Ko YC, Lau LI. Determinants of long-term intraocular pressure after phacoemulsification in primary angle-closure glaucoma. J Glaucoma. 2011;20(9):566-570.

FIGURES LEGEND

FIGURE 1. Prisma flow diagram.

FIGURE 2. The results of the methodological evaluation.

FIGURE 3. Forest plot for mean change in IOP from baseline. SD indicates standard deviation, CI indicates confidence interval.

FIGURE 4. Forest plot for Mean change in PAS. SD indicates standard deviation, CI indicates confidence interval.

FIGURE 5. Forest plot for Mean change in number of glaucoma medications. SD indicates standard deviation, CI indicates confidence interval.

FIGURE 6. Forest plot for Mean change in AOD 500. SD indicates standard deviation, CI indicates confidence interval.

FIGURE 7. Forest plot for Mean change in AOD 750. SD indicates standard deviation, CI indicates confidence interval.

FIGURE 8. Forest plot for Mean change in TSIA 500. SD indicates standard deviation, CI indicates confidence interval.

FIGURE 9. Forest plot for Forest plot for Mean change in TSIA 750. SD indicates standard deviation, CI indicates confidence interval.

FIGURE 10. Forest plot for mean change in BCVA from baseline. SD indicates standard deviation, CI indicates confidence interval.

TABLE 1. Basic characteristics of include studies.

Demographic data of participants in the included studies

Auther (Year)	Study Location	Intervention	Technique (Subgroup)	N	Follow-up (months)	Mean Age	Age (SD)	Male/ Female	Baseline IOP (Mean)	Baseline IOP (SD)	Post-Operative IOP (mean)	Post-Operative IOP (SD)
Angmo D 2019[9]	India	PE+GSL	Instrument	34	6	56.50	9.17	None	30.72	3.88	13.21	1.97
		PE		30		58.77	8.14	Not described	29.48	6.76	13.17	1.66
Husain R 2019[10]	Singapore	PE+GSL	Instrument	38	12	68.1	9.2	13/25	22.9	5.3	15.9	4.5
		PE		40		67.3	8.6	11/29	22.3	8.5	14.3	5.0
Lee CK 2015[11]	Korea	PE+GSL	Instrument	15	2	66	-	11/4	15.87	4.02	13.53	2.80
		PE		15		64	-	14/1	11.33	2.50	11.20	2.54
Rodrigues IA 2017[12]	UK	PE+GSL	Instrument	13	6	67.2	8.4	5/8	27.4	7.2	14.8	2.7
		PE		10		66.1	7.4	5/5	19.6	5.5	14.2	3.1
Shao T 2015[13]	China	PE+GSL	Instrument	23	6	73.61	8.44	None	22.12	5.98	13.65	2.46
		PE		20		69.85	8.56		23.45	7.99	16.40	5.58
Tun TA 2015[14]	Singapore	PE+GSL	Instrument	11	12	66.75	6.53	3/8	21.82	5.81	14.36	2.98
		PE		11		67.77	5.18	2/9	18.73	6.54	16.91	5.72
Eslami Y 2013[15]	USA	PE+GSL	VGP	33	1.5	64.3	9.8	11/16	24.5	6.8	16.9	4.9
		PE		32		65.4	8.4	13/15	21.6	6.0	15.6	5.6
Moghimi S 2015[16]	Iran	PE+GSL	VGP	45	12	61.6	8.3	19/26	23.3	7.3	14.5	2.5
		PE		46		63.2	6.9	19/27	22.3	6.3	14.0	3.7
Varma D 2010[6]	UK	PE+GSL	VGP	25	12	72.40	8.9	16/9	30.12	7.03	13.7	2.89

PE	25	72.96	7.8	8/17	29.68	8.73	16.2	3.55

GSL, goniosynechialysis; VGP, viscogonioplasty; PE, phacoemulsification; IOP, intraocular pressure;

Figure 1

Click here to access/download;Figure;1Flow diagram.tif

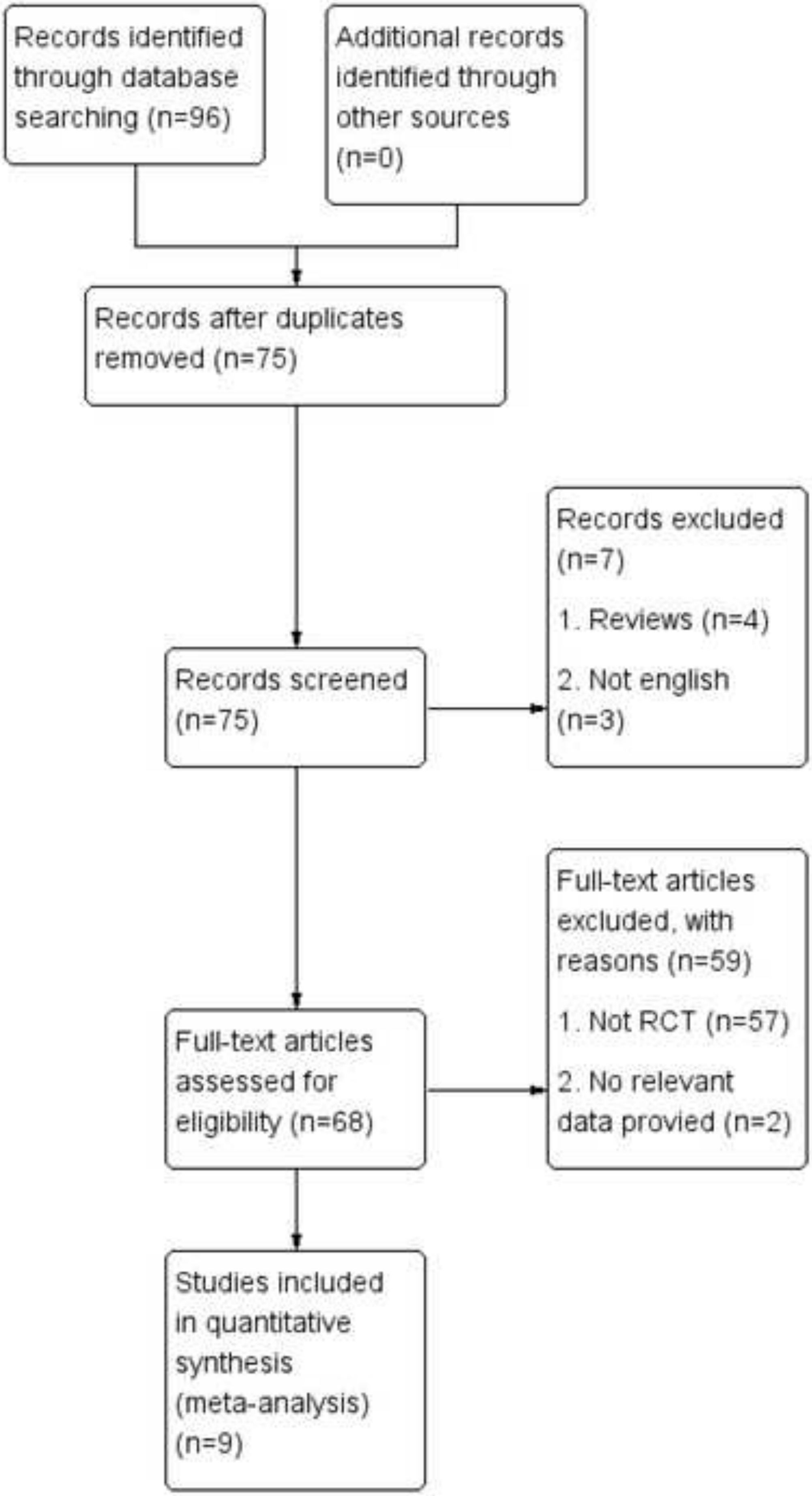

Figure 2 Click here to access/download;Figure;2Risk of bias summary.tif

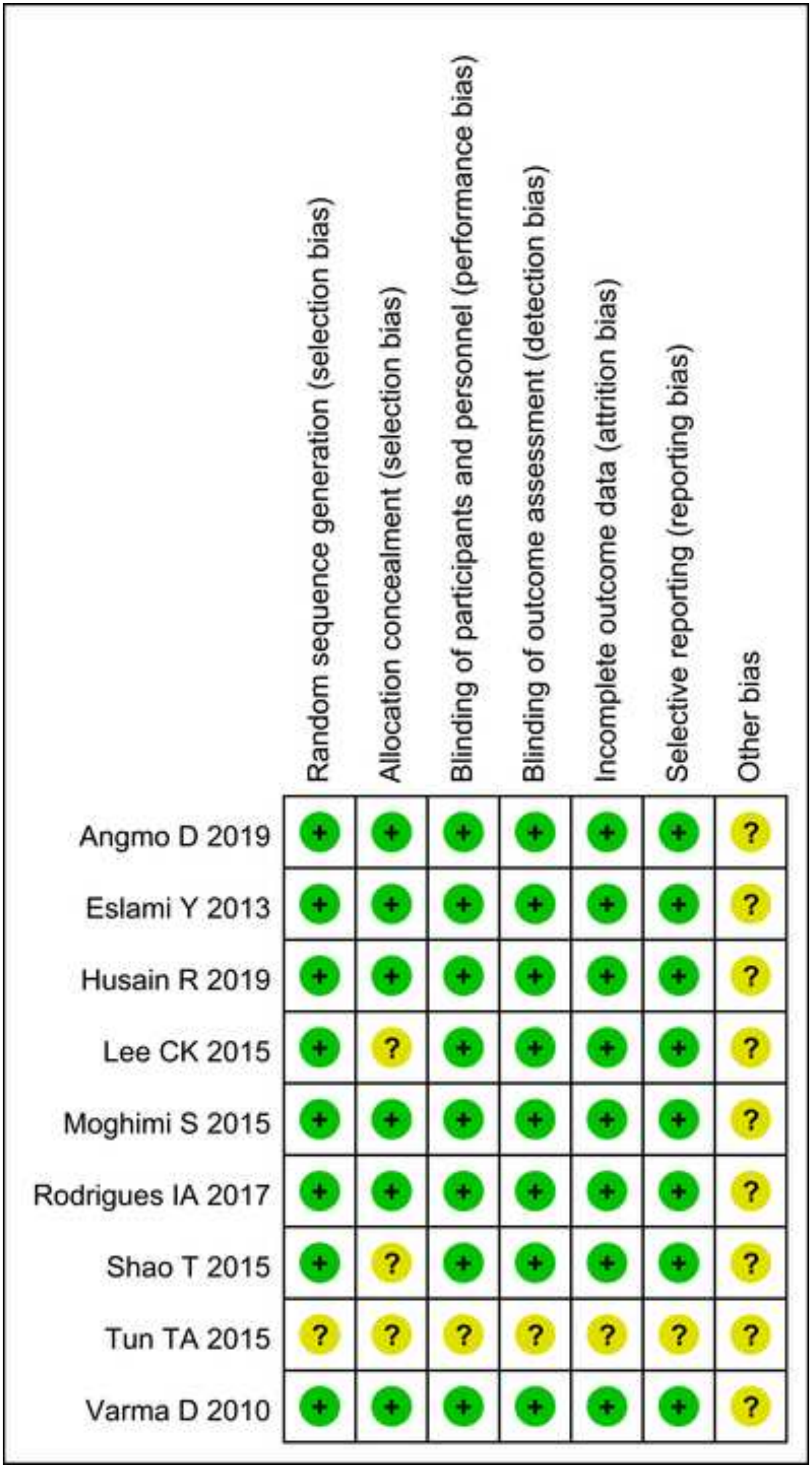

Figure 3

Click here to access/download;Figure;3IOP Forest plot.tif

Study or Subgroup	Phaco+GSL Mean	Phaco+GSL SD	Phaco+GSL Total	Phaco Mean	Phaco SD	Phaco Total	Weight	Mean Difference IV, Random, 95% CI
**1.1.1 Instrument**								
Angmo D 2019	17.51	3.36	34	16.31	6.1	30	18.4%	1.20 [-1.26, 3.66]
Husain R 2019	7.1	6.6	38	7.9	9.4	40	9.7%	-0.80 [-4.39, 2.79]
Lee CK 2015	4.53	4.16	15	2.33	2.38	15	18.8%	2.20 [-0.23, 4.63]
Rodrigues IA 2017	12.6	7.9	13	5.4	4.9	10	4.8%	7.20 [1.94, 12.46]
Shao T 2015	8.47	5.21	23	7.05	7.1	20	8.9%	1.42 [-2.35, 5.19]
Tun TA 2015	7.46	4.87	11	1.82	8.27	11	4.2%	5.64 [-0.03, 11.31]
**Subtotal (95% CI)**			**134**			**126**	**64.9%**	**2.11 [0.27, 3.94]**
Heterogeneity: Tau² = 1.95; Chi² = 8.18, df = 5 (P = 0.15); I² = 39%								
Test for overall effect: Z = 2.25 (P = 0.02)								
**1.1.2 VGP**								
Eslami Y 2013	7.6	6.08	33	6	5.81	32	14.1%	1.60 [-1.29, 4.49]
Moghimi S 2015	8.8	8.4	45	8.3	6.8	46	12.3%	0.50 [-2.64, 3.64]
Varma D 2010	16.42	6.12	25	13.48	7.6	25	8.7%	2.94 [-0.88, 6.76]
**Subtotal (95% CI)**			**103**			**103**	**35.1%**	**1.53 [-0.33, 3.39]**
Heterogeneity: Tau² = 0.00; Chi² = 0.94, df = 2 (P = 0.63); I² = 0%								
Test for overall effect: Z = 1.61 (P = 0.11)								
**Total (95% CI)**			**237**			**229**	**100.0%**	**1.81 [0.62, 3.00]**
Heterogeneity: Tau² = 0.44; Chi² = 9.21, df = 8 (P = 0.32); I² = 13%								
Test for overall effect: Z = 2.98 (P = 0.003)								
Test for subgroup differences: Chi² = 0.19, df = 1 (P = 0.67), I² = 0%								

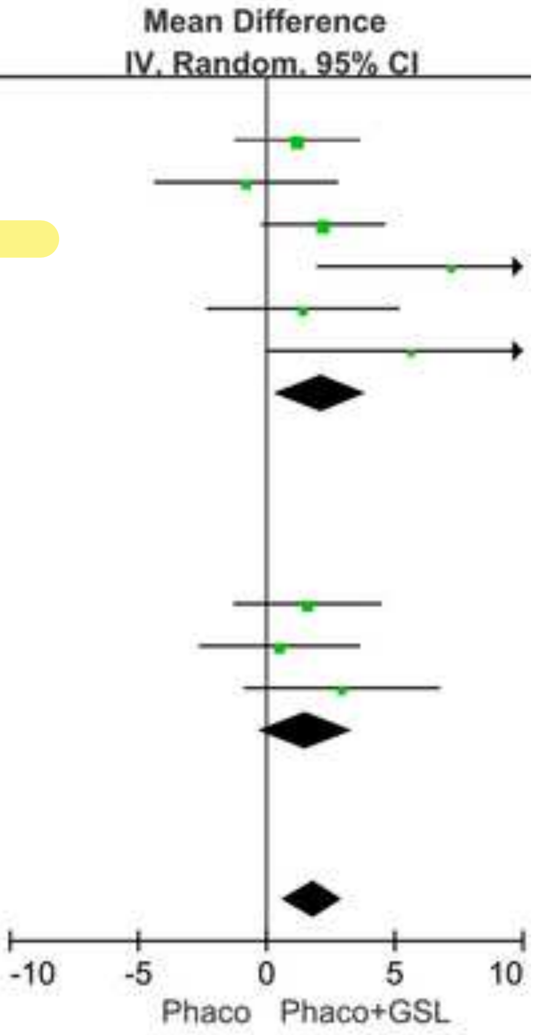

Figure 4

Click here to access/download;Figure;4PAS.tif

Study or Subgroup	Phaco+GSL Mean	Phaco+GSL SD	Phaco+GSL Total	Phaco Mean	Phaco SD	Phaco Total	Weight	Mean Difference IV, Random, 95% CI
**1.4.1 Instrument**								
Husain R 2019	135.9	96.6	38	90.9	117	40	17.8%	45.00 [-2.52, 92.52]
Lee CK 2015	114	90.95	15	118.67	95.38	15	10.2%	-4.67 [-71.36, 62.02]
Rodrigues IA 2017	138.5	101.4	13	22.5	142.5	10	4.5%	116.00 [11.89, 220.11]
Tun TA 2015	111.9	92.1	11	16.5	100.8	11	7.3%	95.40 [14.71, 176.09]
**Subtotal (95% CI)**			**77**			**76**	**39.9%**	**53.32 [5.96, 100.67]**
Heterogeneity: Tau² = 1016.19; Chi² = 5.37, df = 3 (P = 0.15); I² = 44%								
Test for overall effect: Z = 2.21 (P = 0.03)								
**1.4.2 VGP**								
Eslami Y 2013	91.1	75.7	33	32.7	73.7	32	26.2%	58.40 [22.08, 94.72]
Moghimi S 2015	138.2	56.9	45	67.2	84.1	46	33.9%	71.00 [41.55, 100.45]
**Subtotal (95% CI)**			**78**			**78**	**60.1%**	**66.00 [43.13, 88.88]**
Heterogeneity: Tau² = 0.00; Chi² = 0.28, df = 1 (P = 0.60); I² = 0%								
Test for overall effect: Z = 5.66 (P < 0.00001)								
**Total (95% CI)**			**155**			**154**	**100.0%**	**59.15 [36.28, 82.03]**
Heterogeneity: Tau² = 176.06; Chi² = 6.39, df = 5 (P = 0.27); I² = 22%								
Test for overall effect: Z = 5.07 (P < 0.00001)								
Test for subgroup differences: Chi² = 0.22, df = 1 (P = 0.64), I² = 0%								

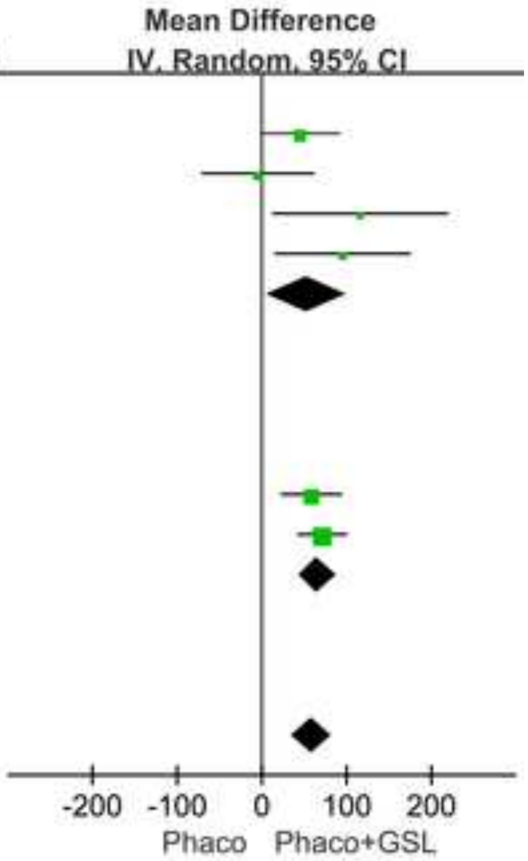

Figure 5

Click here to access/download;Figure;5 Medication Forest plot.tif

	Phaco+GSL			Phaco				Mean Difference
Study or Subgroup	Mean	SD	Total	Mean	SD	Total	Weight	IV, Random, 95% CI
**1.3.1 Instrument**								
Angmo D 2019	1.98	0.44	34	2.18	0.6	30	22.1%	-0.20 [-0.46, 0.06]
Husain R 2019	1.3	1	38	1.7	1	40	15.9%	-0.40 [-0.84, 0.04]
Lee CK 2015	1.2	1.32	15	0.53	0.83	15	8.2%	0.67 [-0.12, 1.46]
Rodrigues IA 2017	0.54	0.78	13	0.3	1.57	10	5.2%	0.24 [-0.82, 1.30]
Shao T 2015	2.975	1.15	23	2.3	1.05	20	10.5%	0.68 [0.02, 1.33]
Tun TA 2015	1.36	1.05	11	1.55	1.04	11	7.1%	-0.19 [-1.06, 0.68]
**Subtotal (95% CI)**			**134**			**126**	**69.0%**	**0.05 [-0.32, 0.42]**
Heterogeneity: Tau² = 0.11; Chi² = 11.74, df = 5 (P = 0.04); I² = 57%								
Test for overall effect: Z = 0.27 (P = 0.79)								
**1.3.2 VGP**								
Eslami Y 2013	1.2	1.06	33	1	1.05	32	13.9%	0.20 [-0.31, 0.71]
Moghimi S 2015	1.3	0.98	45	1.1	0.98	46	17.2%	0.20 [-0.20, 0.60]
**Subtotal (95% CI)**			**78**			**78**	**31.0%**	**0.20 [-0.12, 0.52]**
Heterogeneity: Tau² = 0.00; Chi² = 0.00, df = 1 (P = 1.00); I² = 0%								
Test for overall effect: Z = 1.24 (P = 0.22)								
**Total (95% CI)**			**212**			**204**	**100.0%**	**0.08 [-0.19, 0.35]**
Heterogeneity: Tau² = 0.07; Chi² = 14.09, df = 7 (P = 0.05); I² = 50%								
Test for overall effect: Z = 0.58 (P = 0.56)								
Test for subgroup differences: Chi² = 0.36, df = 1 (P = 0.55), I² = 0%								

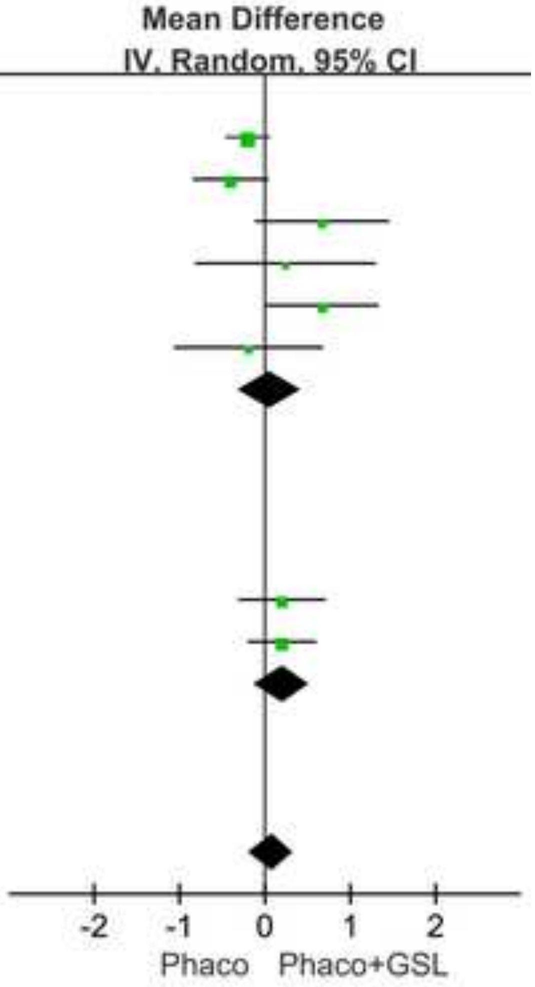

Click here to access/download;Figure;6AOD500.tif

	Phaco+GSL			Phaco				Mean Difference
Study or Subgroup	Mean	SD	Total	Mean	SD	Total	Weight	IV, Random, 95% CI
**1.5.1 Instrument**								
Angmo D 2019	0.3	0.599	34	0.24	0.184	30	29.6%	0.06 [-0.15, 0.27]
Shao T 2015	0.413	0.095	23	0.03	0.026	20	35.3%	0.38 [0.34, 0.42]
**Subtotal (95% CI)**			**57**			**50**	**64.9%**	**0.24 [-0.08, 0.55]**
Heterogeneity: Tau² = 0.05; Chi² = 8.62, df = 1 (P = 0.003); I² = 88%								
Test for overall effect: Z = 1.49 (P = 0.14)								
**1.5.2 VGP**								
Eslami Y 2013	0.205	0.104	33	0.173	0.122	32	35.1%	0.03 [-0.02, 0.09]
**Subtotal (95% CI)**			**33**			**32**	**35.1%**	**0.03 [-0.02, 0.09]**
Heterogeneity: Not applicable								
Test for overall effect: Z = 1.14 (P = 0.26)								
**Total (95% CI)**			**90**			**82**	**100.0%**	**0.16 [-0.12, 0.45]**
Heterogeneity: Tau² = 0.06; Chi² = 104.42, df = 2 (P < 0.00001); I² = 98%								
Test for overall effect: Z = 1.14 (P = 0.25)								
Test for subgroup differences: Chi² = 1.61, df = 1 (P = 0.20), I² = 37.9%								

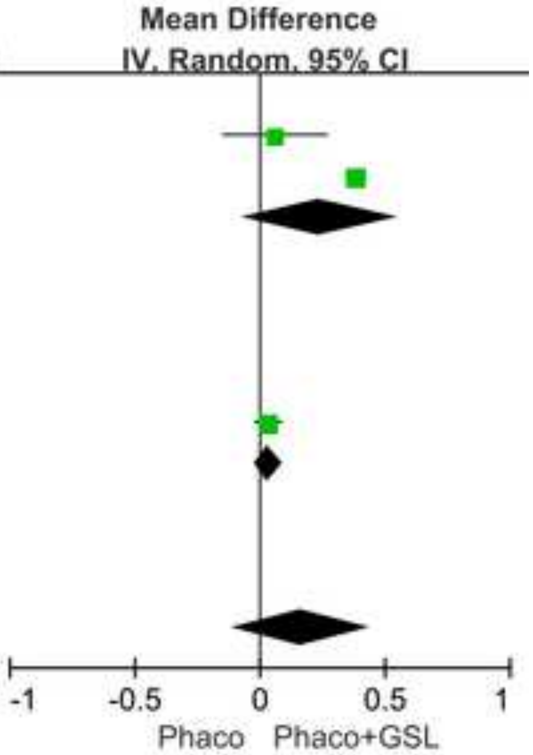

Figure 7 Click here to access/download;Figure;7AOD750.tif

Study or Subgroup	Phaco+GSL Mean	Phaco+GSL SD	Phaco+GSL Total	Phaco Mean	Phaco SD	Phaco Total	Weight	Mean Difference IV, Random, 95% CI
**1.6.1 Instrument**								
Angmo D 2019	0.34	0.689	34	0.35	0.261	30	31.3%	-0.01 [-0.26, 0.24]
Shao T 2015	0.62	0.151	23	0.027	0.046	20	34.4%	0.59 [0.53, 0.66]
**Subtotal (95% CI)**			**57**			**50**	**65.7%**	**0.30 [-0.29, 0.89]**
Heterogeneity: Tau² = 0.17; Chi² = 20.98, df = 1 (P < 0.00001); I² = 95%								
Test for overall effect: Z = 1.01 (P = 0.31)								
**1.6.2 VGP**								
Eslami Y 2013	0.325	0.127	33	0.296	0.169	32	34.3%	0.03 [-0.04, 0.10]
**Subtotal (95% CI)**			**33**			**32**	**34.3%**	**0.03 [-0.04, 0.10]**
Heterogeneity: Not applicable								
Test for overall effect: Z = 0.78 (P = 0.44)								
**Total (95% CI)**			**90**			**82**	**100.0%**	**0.21 [-0.23, 0.66]**
Heterogeneity: Tau² = 0.15; Chi² = 135.76, df = 2 (P < 0.00001); I² = 99%								
Test for overall effect: Z = 0.93 (P = 0.35)								
Test for subgroup differences: Chi² = 0.82, df = 1 (P = 0.36), I² = 0%								

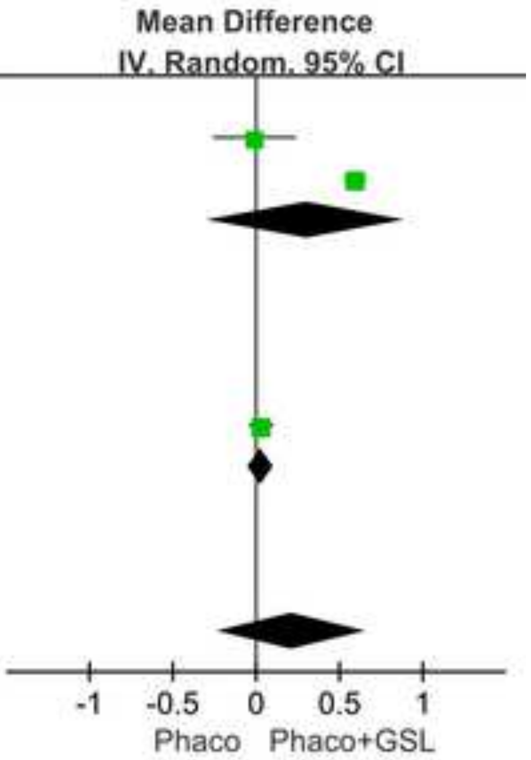

Figure 8

Click here to access/download;Figure;8TISA500.tif

Study or Subgroup	Phaco+GSL Mean	Phaco+GSL SD	Phaco+GSL Total	Phaco Mean	Phaco SD	Phaco Total	Weight	Mean Difference IV, Random, 95% CI
**1.7.1 Instrument**								
Angmo D 2019	0.1	0.087	34	0.08	0.053	30	24.2%	0.02 [-0.01, 0.05]
Shao T 2015	0.138	0.03	23	0.012	0.01	20	25.6%	0.13 [0.11, 0.14]
**Subtotal (95% CI)**			**57**			**50**	**49.8%**	**0.07 [-0.03, 0.18]**
Heterogeneity: Tau² = 0.01; Chi² = 31.18, df = 1 (P < 0.00001); I² = 97%								
Test for overall effect: Z = 1.40 (P = 0.16)								
**1.7.2 VGP**								
Eslami Y 2013	0.063	0.033	33	0.055	0.042	32	25.3%	0.01 [-0.01, 0.03]
Moghimi S 2015	0.054	0.033	45	0.02	0.084	46	24.9%	0.03 [0.01, 0.06]
**Subtotal (95% CI)**			**78**			**78**	**50.2%**	**0.02 [-0.01, 0.04]**
Heterogeneity: Tau² = 0.00; Chi² = 2.54, df = 1 (P = 0.11); I² = 61%								
Test for overall effect: Z = 1.50 (P = 0.13)								
**Total (95% CI)**			**135**			**128**	**100.0%**	**0.05 [-0.02, 0.12]**
Heterogeneity: Tau² = 0.00; Chi² = 128.91, df = 3 (P < 0.00001); I² = 98%								
Test for overall effect: Z = 1.37 (P = 0.17)								
Test for subgroup differences: Chi² = 1.02, df = 1 (P = 0.31), I² = 1.7%								

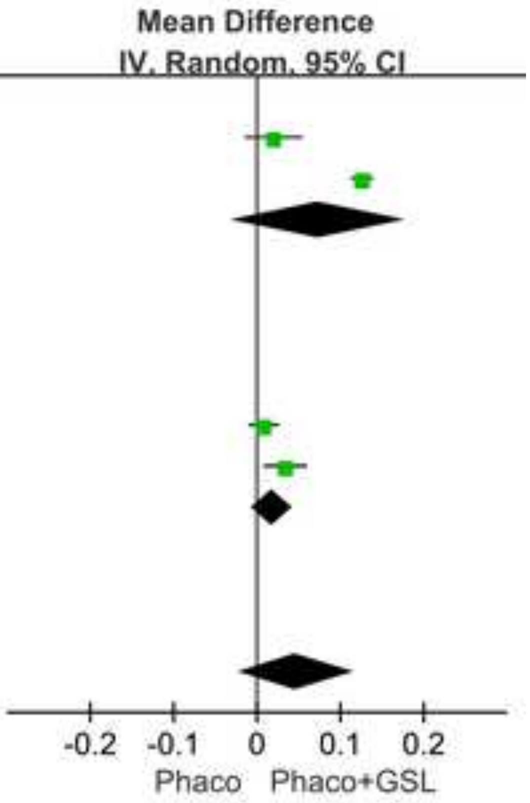

Figure 9

Click here to access/download;Figure;9TISA750.tif

Study or Subgroup	Phaco+GSL Mean	Phaco+GSL SD	Phaco+GSL Total	Phaco Mean	Phaco SD	Phaco Total	Weight	Mean Difference IV, Fixed, 95% CI
**1.8.1 Instrument**								
Angmo D 2019	0.17	0.358	34	0.15	0.104	30	1.2%	0.02 [-0.11, 0.15]
Shao T 2015	0.27	0.053	23	0.023	0.017	20	35.4%	0.25 [0.22, 0.27]
Tun TA 2015	0.1	0.036	11	0.09	0.047	11	15.2%	0.01 [-0.02, 0.04]
**Subtotal (95% CI)**			**68**			**61**	**51.8%**	**0.17 [0.15, 0.19]**
Heterogeneity: Chi² = 129.14, df = 2 (P < 0.00001); I² = 98%								
Test for overall effect: Z = 17.83 (P < 0.00001)								
**1.8.2 VGP**								
Eslami Y 2013	0.126	0.063	33	0.115	0.074	32	16.6%	0.01 [-0.02, 0.04]
Moghimi S 2015	0.119	0.061	45	0.084	0.057	46	31.6%	0.03 [0.01, 0.06]
**Subtotal (95% CI)**			**78**			**78**	**48.2%**	**0.03 [0.01, 0.05]**
Heterogeneity: Chi² = 1.30, df = 1 (P = 0.26); I² = 23%								
Test for overall effect: Z = 2.67 (P = 0.008)								
**Total (95% CI)**			**146**			**139**	**100.0%**	**0.10 [0.09, 0.12]**
Heterogeneity: Chi² = 239.83, df = 4 (P < 0.00001); I² = 98%								
Test for overall effect: Z = 14.69 (P < 0.00001)								
Test for subgroup differences: Chi² = 109.39, df = 1 (P < 0.00001), I² = 99.1%								

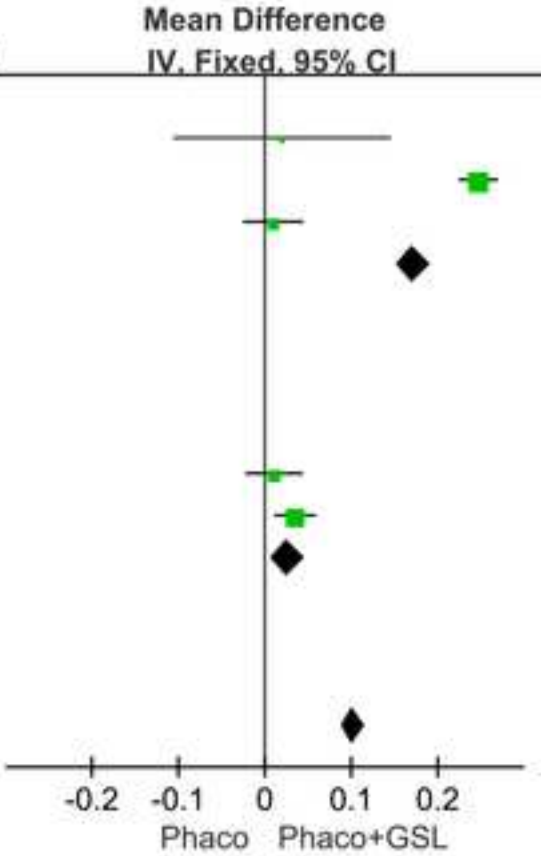

Study or Subgroup	Phaco+GSL Mean	Phaco+GSL SD	Phaco+GSL Total	Phaco Mean	Phaco SD	Phaco Total	Weight	Mean Difference IV, Random, 95% CI
**1.2.1 Instrument**								
Angmo D 2019	0.32	0.14	34	0.27	0.18	30	49.4%	0.05 [-0.03, 0.13]
Lee CK 2015	0.09	0.44	15	0.19	0.24	15	4.9%	-0.10 [-0.35, 0.15]
Rodrigues IA 2017	0.1	0.36	13	0.07	0.46	10	2.6%	0.03 [-0.32, 0.38]
Shao T 2015	0.698	0.57	23	0.47	0.28	20	4.5%	0.23 [-0.04, 0.49]
**Subtotal (95% CI)**			**85**			**75**	**61.4%**	**0.05 [-0.03, 0.13]**
Heterogeneity: Tau² = 0.00; Chi² = 3.11, df = 3 (P = 0.37); I² = 4%								
Test for overall effect: Z = 1.26 (P = 0.21)								
**1.2.2 VGP**								
Eslami Y 2013	0.29	0.28	33	0.28	0.31	32	15.2%	0.01 [-0.13, 0.15]
Moghimi S 2015	0.21	0.31	45	0.22	0.25	46	23.4%	-0.01 [-0.13, 0.11]
**Subtotal (95% CI)**			**78**			**78**	**38.6%**	**-0.00 [-0.09, 0.09]**
Heterogeneity: Tau² = 0.00; Chi² = 0.05, df = 1 (P = 0.83); I² = 0%								
Test for overall effect: Z = 0.05 (P = 0.96)								
**Total (95% CI)**			**163**			**153**	**100.0%**	**0.03 [-0.03, 0.09]**
Heterogeneity: Tau² = 0.00; Chi² = 3.96, df = 5 (P = 0.56); I² = 0%								
Test for overall effect: Z = 1.05 (P = 0.29)								
Test for subgroup differences: Chi² = 0.74, df = 1 (P = 0.39), I² = 0%								

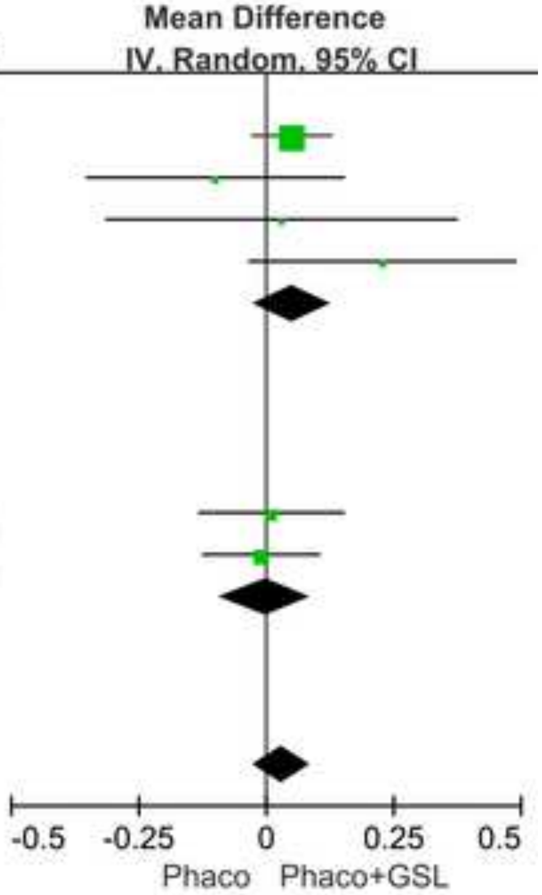

Prisma checklist

Click here to access/download
Supporting Information
PRISMA_2020_checklist.docx
