# Supplementary material for: Phacoemulsification combined with goniosynechialysis versus phacoemulsification alone for patients with primary angle‑closure disease: A meta-analysis
Source: PLoS One. 2024 Feb 2;19(2):e0296849. doi: 10.1371/journal.pone.0296849 (PMC10836673; doi:10.1371/journal.pone.0296849)
Supplement: Attachment — Submitted filename: response1028.docx [file pone.0296849.s003.docx]

Dear reviewer,

Thank you for your comments. The following is our replies.

For reviewer #1:

Congratulations for your effort in analyzing the current data related to this surgical procedure. There are some important issues that should be addressed before this paper is published:

1. Demographic data of the participants should be discussed. In the majority, this studies were conducted in asian populations. It is largely known that can have different patterns of anatomic characteristics in this population when compared to caucasian or hispanic origin population.

Answer: We added limitations of this study in the discussion section.

2. I have doubts about the baseline and postoperative mean IOP used to perform the metanalysis (Figure 3), and because of that, maybe conclusion can be different.

Answer: In Figure 3, mean IOP was the change of IOP before and after surgery.

3. Authors delineated conclusions before discuss the heterogeneity of the compiled data.

Answer: Generally, if I^2^ was higher than 50%, indicating a high degree of heterogeneity, a random-effect model should be applied. If I^2^ was lower than 50%, indicating a low-to-moderate heterogeneity, both random-effect model and fixed-effect model could be applied. In our meta-analysis, we used a random-effect model for both low-to-moderate and high degree of heterogeneity. Therefore, we did not discuss the heterogeneity in the manuscript.

For Reviewer #2:

1. Page 2, line 17: TSIA must be corrected to TISA.

Answer: We revised it.

2. Page 5, line 3: Which device(s) was/were applied for the secondary outcome, eg, UBM, AS-OCT?

Answer: Only AS-OCT was applied for AOD and TISA in the included studies. We added it in the method section.

3. P7, line 20: TSIA must be corrected to TISA.

Answer: We revised it.

4. Discussion: Limitations of this meta-analysis should be discussed.

Answer: We added limitations of this study in the discussion section.

5. P11: References 18 and 20 are not related to this meta-analysis study. Why did you cited them?

Answer: We have referred to the research methods of these articles, so we have cited them.

For Reviewer #3:

First of all, thank you for your contribution in field of glaucoma.

This manuscript entitled 'Phacoemulsification combined with goniosynechialysis versus phacoemulsification alone for patients with primary angle-closure disease: a meta-analysis" were well written. This manuscript well summarized the pre-existing studies and results.

In general, this article well adhere to PRISMA 2020. In specific the title identify the report as meta-analysis and methods included the Cochrane review (RevMan 5.4). Introduction well describe the rationale for the review. Methods specified the inclusion and exclusion criteria well, and selection process looks like clear and Interpretation is not clinically biased.

(In aspect of Search strategy, Selection process, data collection process, Study risk of Bias assessment)

Although some included study has small sample size, the interpretation looks like technically sound. And the assessment of bias is adequate.

If possible, the explain the detail of benefits and possible risk of Phaco+GSL for PAC for general readers.

Thank you again for your contribution for meta-analysis for Phaco + GSL.

Answer: GSL is a safe surgical procedure. Compared with Phaco alone, the possible risk of Phaco+GSL is hyphema, which was reported in three included studies [9, 12, 13]. We have added the above content to the discussion section.

Best regards.

Sincerely

Haiqing Bai
